# The Mechanisms Underlying Chronic Inflammation in Rheumatoid Arthritis from the Perspective of the Epigenetic Landscape

**DOI:** 10.1155/2016/6290682

**Published:** 2016-12-28

**Authors:** Yasuto Araki, Toshihide Mimura

**Affiliations:** ^1^Department of Rheumatology and Applied Immunology, Faculty of Medicine, Saitama Medical University, Saitama, Japan; ^2^Project Research Division, Research Center for Genomic Medicine, Saitama Medical University, Saitama, Japan

## Abstract

Rheumatoid arthritis (RA) is a chronic inflammatory autoimmune disease that is characterized by synovial hyperplasia and progressive joint destruction. The activation of RA synovial fibroblasts (SFs), also called fibroblast-like synoviocytes (FLS), contributes significantly to perpetuation of the disease. Genetic and environmental factors have been reported to be involved in the etiology of RA but are insufficient to explain it. In recent years, accumulating results have shown the potential role of epigenetic mechanisms, including histone modifications, DNA methylation, and microRNAs, in the development of RA. Epigenetic mechanisms regulate chromatin state and gene transcription without any change in DNA sequence, resulting in the alteration of phenotypes in several cell types, especially RASFs. Epigenetic changes possibly provide RASFs with an activated phenotype. In this paper, we review the roles of epigenetic mechanisms relevant for the progression of RA.

## 1. Introduction

Rheumatoid arthritis (RA) is a chronic autoimmune inflammatory disease that results in progressive destruction of articular cartilage and bone and is hard to treat effectively [1]. RA is two- to fourfold more common in women than in men and affects approximately 1% of the world's population [2]. The pathogenesis of this disease is not yet completely understood as it likely has a complex, multifactorial etiology. Anticitrullinated peptide/protein antibodies (ACPA) were found to be autoantibodies specific for RA [3]. Citrullination is the posttranslational modification of arginine into citrulline by peptidylarginine deiminases (PAD) [4]. Variable citrullinated autoantigens that are recognized by ACPA, such as keratin, filaggrin, fibrin/fibrinogen, vimentin, type II collagen, cartilage oligomeric matrix protein (COMP), and *α*-enolase, have been identified in RA [5–11]. However, each of these autoantigens is present only in a particular subset of RA patients, suggesting that RA is a syndrome, not a disease. Because citrulline is a nonstandard amino acid, the citrullination of specific antigens could promote the generation of neoepitopes that are recognized by CD4^+^ T cells in RA patients. Autoreactive CD4^+^ T cells have been observed in certain animal models, such as adjuvant arthritis in rats [12]. Since CD4^+^ T cells in RA synovial fluid are oligoclonal, the CD4^+^ T cell activation process is thought to be antigen driven [13, 14]. However, the oligoclonality of CD4^+^ T cells has actually been demonstrated in only a few RA patients. Therefore, the role of autoreactive CD4^+^ T cells in the pathogenesis of RA is not yet entirely convincing. The degree of macrophage infiltration into the synovium is correlated with the degree of bone erosion in the affected joints in RA [15]. CD5^+^ B cells in the synovium produce nonspecific antibodies, such as IgM/IgG/IgA rheumatoid factors (RF) [16, 17]. This production is induced by interleukin- (IL-) 10 in RA [18]. On the other hand, the production of ACPA requires the help of CD4^+^ T cells. Thus, it is obvious that macrophages and B cells play an important role in the pathogenesis of RA, but the contribution of CD4^+^ T cells to their activation is still controversial. The proinflammatory cytokines, such as tumor necrosis factor *α* (TNF*α*), IL-1*β*, and IL-6, are produced by activated macrophages and stimulate the synovial fibroblasts (SFs), also called fibroblast-like synoviocytes (FLS), that play a critical role in the joint destruction that occurs in RA [19].

In the present paradigm, it is presumed that RA is triggered in genetically predisposed individuals by exposure to environmental factors. Furthermore, environmental factors are associated with epigenetic changes. Epigenetic regulation has been the current focus of many studies because it is a novel and attractive area. In this review, we summarize the recent progress that has been made in the understanding of diverse epigenetic mechanisms involved in the pathogenesis of RA, with an emphasis on RASFs.

## 2. The Pathogenesis of RA

### 2.1. RASFs

The synovial lining layer of joints is two to three cells thick and consists of SFs and synovial macrophages. In RA, the lining layer undergoes dramatic hyperplasia and increases to a density of 10 to 15 cells thick [20, 21]. At the articular borders, the lining layer forms a pannus that invades the adjacent articular cartilage and subchondral bone. The sublining layer has fewer SFs and synovial macrophages in a loose tissue matrix. The sublining layer also undergoes dramatic hyperplasia and is infiltrated with immune cells [22]. Synovial tissues were found to be enriched with memory CD45RO^+^ T cells, most of which were not activated T cells but rather mature memory T cells that exhibited an enhanced capacity for transendothelial migration [23]. The expression of chemokine receptor C-X-C motif chemokine receptor 4 (CXCR4) was highly expressed in synovial memory T cells [24]. Synovial T cells are thought to be attracted by chemokines and receive survival signals such as IL-7 and IL-15 [25, 26]. Since SFs maintain an activated and aggressive phenotype with a tumor-like behavior in RA, they play a central role in joint destruction and persistent inflammation in RA [27]. RASFs show an increased capacity to migrate and produce matrix-degrading enzymes, such as matrix metalloproteinases (MMPs) and cathepsins, which contribute to cartilage destruction. Their increased proliferation and resistance to apoptosis are controversial [20]. The pannus formation is composed of infiltrating cells, such as monocytes/macrophages, as well as RASFs. RASFs secrete proinflammatory cytokines and chemokines that perpetuate inflammation. In addition, RASFs produce receptor activator of nuclear factor-kappa B ligand (RANKL) and promote osteoclast differentiation, resulting in bone destruction [28].

**Figure 1:**
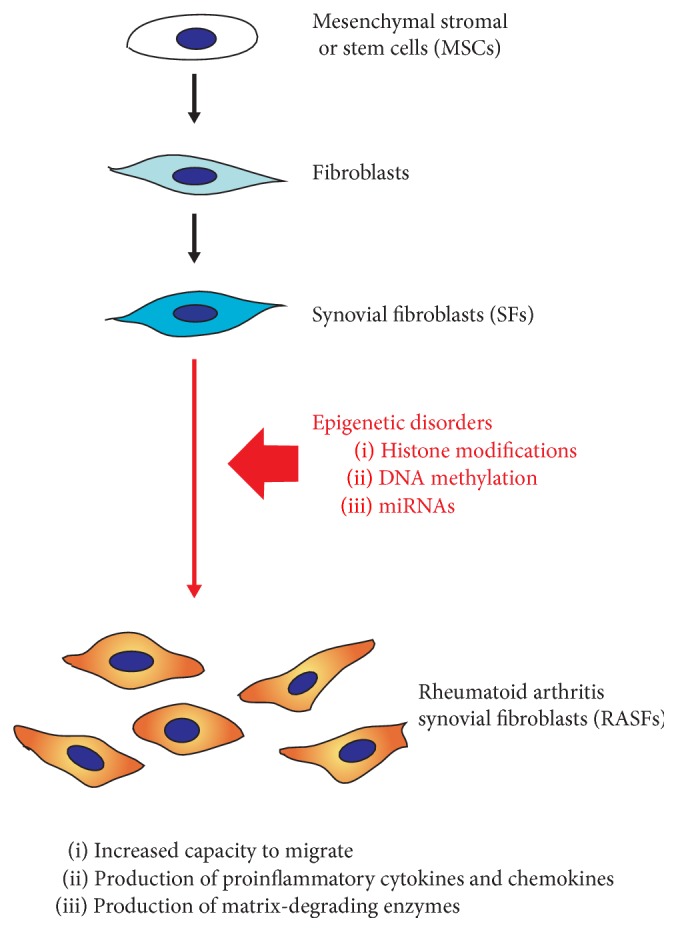
Epigenetic disorders induce the activation of rheumatoid arthritis synovial fibroblasts (RASFs). Normal SFs are differentiated from mesenchymal stromal or stem cells (MSCs) under normal epigenetic regulation in noninflammatory joints of healthy individuals. The activation of SFs is caused by aberrant epigenetic changes in inflammatory joints of RA.

Understanding the mechanisms underlying the activation of RASFs may lead to the development of the most suitable RA therapy. Recent advances have suggested that not only genetic and environmental factors, but also epigenetic changes, are implicated in the pathogenesis of RA [29, 30]. Mesenchymal stromal or stem cells (MSCs) differentiate into normal SFs under normal epigenetic regulation. The activation of SFs can be caused by altered profiles of gene expression that result from epigenetic dysregulation in RA (Figure 1).

### 2.2. Genetic Factors in RA

Multiple lines of evidence have revealed that genetic factors participate in the etiology of RA. The pairwise concordance rate for RA was 12.3% in monozygotic (MZ) twins and 3.5% in dizygotic (DZ) twins in a Finnish population [31]. Another group showed that the concordance rate for RA was 15.4% in MZ twins and 3.6% in DZ twins in a United Kingdom (UK) population [32]. According to these twin studies, the concordance of MZ twins was higher than that of DZ twins in RA. Analysis of these studies showed that the heritability of RA was 65% in the Finnish group and 53% in the UK group [33]. The genetic contribution was not affected by sex, age, age at onset, or disease severity. A family study demonstrated that the standardized incidence ratio (relative risk [RR]) for RA was 3.02 in the offspring of affected parents, 4.64 in siblings, 9.31 in multiplex families, 6.48 in twins, and 1.17 in spouses [34]. Serological studies reported that susceptibility to RA was associated with certain human leukocyte antigen- (HLA-) DRB1 alleles that contain conserved five-amino-acid-sequence motifs QKRAA/QRRAA/RRRAA, termed shared epitope (SE) [35]. SE-coding HLA-DRB1 alleles include HLA-DRB1*∗*0401, *∗*0404, and *∗*0101 and are associated with RA severity [36]. In addition, SE alleles are strongly associated with the production of ACPA in RA [37]. Candidate gene approaches, genome-wide association studies (GWAS), and trans-ethnic GWAS meta-analyses have identified a number of RA risk genes, such as HLA-DRB1, PTPN22, STAT4, CCR6, TNFAIP3, PADI4, CD40, and FCRL3, many of which are involved in the functions of immune cells, including T cells, B cells, and macrophages [38, 39].

### 2.3. Environmental Factors in RA

It is undeniable that genetic factors play an important role in the pathogenesis of RA; however, environmental factors also trigger the development of this disease. For example, cigarette smoking influences both the incidence and severity of RA in a dose-dependent fashion and also increases the risk of ACPA production [40]. The RR of RA by cigarette smoking is about 1.8 [41]. However, smokers are subject to the development of periodontitis.* Porphyromonas gingivalis* is a major causative pathogen of periodontitis and expresses its own unique enzyme,* Porphyromonas gingivalis* peptidylarginine deiminase (PPAD), which has PAD activity and catalyzes citrullination [42]. The periodontitis-induced citrullination generates autoantigens that drive autoimmunity and induce the production of ACPA in RA [43]. It is possible that smoking-related periodontitis, but not smoking itself, is a direct environmental trigger of the development of RA. On the other hand, smoking has been reported to promote citrullination in the lungs, mediated by PAD enzymes from smoking-activated phagocytes [44, 45]. Smoking associated inflammatory events in the lung are potential environmental triggers for both ACPA production and RA development. Although the precise pathogenic effect of smoking in RA remains unknown, several mechanisms, including periodontitis-induced or lung inflammation-induced citrullination, have been proposed to explain how smoking plays an important role in RA pathogenesis. Molecular mimicry as a result of Epstein-Barr virus (EBV), a ubiquitous virus, may trigger RA [46], as antibodies against the EBV-encoded proteins cross-react with RA-specific proteins [47, 48]. In addition, EBV DNA loads in peripheral blood mononuclear cells, saliva, and synovium increased in RA [49–51]. High numbers of circulating EBV-infected B cells have been observed in RA [52], and EBV-specific T cell function was impaired in this disease [53]. Although several reports have shown aberrant immune responses to EBV in RA, it remains unknown whether these abnormalities are causative. Estrogen enhances the immune response, whereas androgen and progesterone suppress it [54]. Low androgen levels, high estrogen levels, and a reduced ratio of androgen/estrogen have been observed in male and female RA patients, and androgen replacement therapy was shown to improve disease activity in male RA patients [55]. Since vitamin D represses the development of autoimmunity in experimental animal models, it is expected to have immunomodulatory effects [56]. A greater intake of vitamin D reduced the incidence of RA in older woman [57]. Exposure to silica through the respiratory tract increased the risk of developing RA [58].

## 3. Epigenetic Mechanisms

### 3.1. Chromatin and Epigenetic Mechanisms

In 1942, the British developmental biologist Conrad Hal Waddington first used the term “epigenetics” derived from the Greek word “epigenesis” in his* Principles of Embryology* textbook. The epigenetic landscape theory that he proposed described a process in which gene regulation (e.g., mutations) modulates development. Recently epigenetics has been defined as “a stably heritable phenotype resulting from changes in a chromosome without alterations in the DNA sequence” [59]. Epigenetic information is transmitted through either mitosis or meiosis.

DNA is highly packaged into chromatin in the nucleus of eukaryotic cells [60]. The basic subunits of the chromatin are nucleosomes consisting of two copies each of the core histone proteins H2A, H2B, H3, and H4; the DNA wrapped around the core contains 146-147 base pairs. There are two basic forms of chromatin structures: (1) euchromatin is an open chromatin structure in which DNA-binding proteins, such as transcription factors (TFs), are accessible to DNA, resulting in active transcription; and (2) heterochromatin is a condensed chromatin structure that lacks accessibility to the transcriptional machinery, resulting in gene silencing.

Several epigenetic mechanisms, including posttranslational histone modifications, DNA methylation, and microRNAs (miRNAs), determine the specific chromatin structure, consequently influencing gene transcription without altering the DNA sequence itself [61]. Chromatin structure in DNA-regulating regions, such as promoters and enhancers, regulates gene transcription by altering the accessibility for TFs.

### 3.2. Histone Modifications

Covalent posttranslational modifications in histone N-terminal tails, including acetylation, methylation, ubiquitination, and phosphorylation, control the chromatin state and gene transcription [62]. Each modification has specific functions [63, 64]. Active histone markers that are associated with euchromatin and gene activation include acetylation of H2A, H2B, H3 lysine 9 (H3K9), H3K14, H4K5, and H4K16; methylation of H2BK5, H3K4, H3K36, and H3K79; phosphorylation of H3 threonine 3 (H3T3), H3 serine 10 (H3S10), H3S28, and H4S1; and ubiquitination of H2BK120. On the other hand, repressive histone markers that are correlated with heterochromatin and gene repression include methylation of H3K9, H3K27, and H4K20; ubiquitination of H2AK119; and sumoylation of H2AK126, H2BK6, and H2BK7.

Among these modifications, acetylation and methylation have been intensively studied. Histone acetyltransferases (HATs) transfer acetyl groups to lysine residues, resulting in gene activation, whereas histone deacetylases (HDACs) remove acetyl groups, resulting in gene silencing [65]. Histone methyltransferases (HMTs) transfer methyl groups, whereas histone demethylases (HDMs) remove methyl groups [66]. HMTs and HDMs specifically catalyze particular residues. The functions of histone methylation are affected by both the position of the residue and the number of methyl groups. According to the histone code hypothesis, multiple histone modifications, acting in a combinatorial or sequential fashion on one or multiple histone tails, specify unique downstream functions [67]. A complex combination of these histone modifications is thought to regulate chromatin structure and gene transcription.

### 3.3. DNA Methylation

DNA methylation is a biochemical process in which a methyl group is added to a carbon 5′ position of a CpG dinucleotide and 5-methylcytosine (5mC) is generated [68]. This process occurs in regions of clustered CpG dinucleotides, known as CpG islands, which are typically located in the promoters of genes [69]. Approximately 70% of annotated gene promoters are correlated with CpG islands [70]. A high level of DNA methylation at CpG islands inhibits binding of TFs and represses gene transcription, whereas a low level is associated with an open chromatin structure and active gene transcription [71]. Although some CpG islands are located at a distance from promoters, they can also affect gene transcription [72].

The process of DNA methylation is catalyzed by DNA methyltransferases (DNMTs), including DNMT1, DNMT3a, and DNMT3b, which use S-adenosylmethionine (SAM) as the methyl donor [73]. DNMT1 maintains the DNA methylation patterns through cell replication [74]. Specifically, it is upregulated during the S phase of the cell cycle, is recruited to DNA replication forks, and methylates CpG sites on daughter strands. On the other hand, DNMT3a and DNMT3b are de novo methyltransferases and establish methylation patterns in both unmethylated and hemimethylated CpG sites with equal efficiencies [75].

Recent advances have revealed the process of active DNA demethylation by the ten-eleven translocation (TET) family of enzymes, including TET1, TET2, and TET3, which are *α*-ketoglutarate- and Fe(II)-dependent dioxygenases and catalyze the conversion of 5mC to 5-hydroxymethylcytosine (5hmC) [76]. It has been demonstrated that TET proteins contribute to the additional oxidation of 5hmC to 5-formylcytosine (5fC) and 5-carboxylcytosine (5caC) [77]. An unmodified C is generated either through a replication-dependent dilution of 5hmC or through the removal of 5fC or 5caC by thymine DNA glycosylate- (TDG-) mediated base excision repair [78].

### 3.4. miRNAs

miRNAs are short noncoding RNAs that are 19–25 nucleotides long and cause posttranscriptional and posttranslational gene silencing [79]. The miRNA sequence is transcribed to long primary miRNA (pri-miRNA) of several kb in length that are capped and polyadenylated by RNA polymerase II and then processed by Drosha to form an approximately 70-nucleotide hairpin precursor miRNA (pre-miRNA) in the nucleus. The pre-miRNA is processed in the cytosol by RNase III-type enzyme Dicer to a mature miRNA duplex of approximately 22 nucleotides. The double-stranded miRNA complex is associated with the RNA-induced silencing complex (RISC), which is composed of the transactivation-responsive RNA-binding protein (TRBP) and Argonaute (Ago2). After the complementary strand is removed from the RISC, functional miRNA binds to the 3′-untranslated region (UTR) of the messenger RNA (mRNA) of a target gene and causes mRNA cleavage or translational repression [80, 81]. Perfect binding between miRNA and the mRNA target results in the Ago-catalyzed cleavage of the mRNA strand, whereas imperfect binding leads to the repression of mRNA translation. Although to date approximately 1900 human miRNAs have been identified, most of their target genes remain unknown.

### 3.5. Chromatin Structure-Based Regulation of Gene Transcription

Epigenetic mechanisms regulate chromatin structure and the sustained and distinct patterns of gene expression through cell differentiation. Complex is the association between chromatin structure and gene transcription. Analyses of genome-wide profiles of histone methylation and gene expression have demonstrated a general correlation between histone methylation patterns and gene expression [82]. The levels of trimethylation at H3K4 (H3K4me3) and at H3K27 (H3K27me3) are positively and negatively correlated with gene expression, respectively. These correlations demonstrate four distinct states: repressed, active, poised, and bivalent [83, 84]. In the repressed state, the gene locus has a condensed chromatin structure and gene transcription is repressed. In the active state, the gene locus has an open chromatin structure and gene transcription is active. In the poised state, the gene locus has an open chromatin structure, similar to the active state, but there is no active gene transcription at resting. However, following activation, gene transcription can be rapidly activated. Genes in the bivalent state contain high levels of both active and repressive histone markers. The chromatin structure can change to an open or condensed state both through cell differentiation and upon activation. The specific epigenetic landscape provides the chromatin basis for distinct gene transcription.

## 4. Epigenetic Abnormalities in RASFs

### 4.1. Histone Modifications in RASFs

Aberrant histone modifications have been shown to be involved in the activation of RASFs (Table 1). An H3K27-specific HMT, enhancer of zeste homologue 2 (EZH2), was highly expressed in RASFs and induced by TNF*α* through nuclear factor-kappa B (NF-*κ*B) and Jun kinase pathways [85]. Secreted fizzled-related protein 1 (SFRP1), an inhibitor of Wnt signaling, was identified as the target gene and was associated with the activation of RASFs. In addition, its expression was found to be associated with specific histone markers in the promoter, such as H3K4me3 and H3K27me3. T-box transcription factor 5 (TBX5) is highly expressed in RASFs [86]. Correspondingly, active histone markers, including H3K4me3 and histone acetylation, were increased in the TBX5 promoter of RASFs. Overexpression of TBX5 altered expression of 790 genes, including IL-8, C-X-C motif ligand 12 (CXCL12), and C-C motif ligand 20 (CCL20). It has been suggested that TBX5 is newly identified as an inducer of important chemokines in RASFs. MMP-1, MMP-3, MMP-9, and MMP-13, which have pivotal roles in the pathogenesis of RA, are highly expressed in RASFs [87]. Accordingly, the levels of H3K4me3 increased, whereas those of H3K27me3 decreased in the MMP promoters in RASFs. WD (tryptophan-aspartate) repeat domain 5 (WDR5) is a core subunit of complex proteins associated with SET1 (COMPASS) or COMPASS-like complexes that catalyze H3K4 methylation, which is necessary for the generation of H3K4me3. WDR5 knockdown reduced H3K4me3 as well as the expression of MMP-1, MMP-3, MMP-9, and MMP-13 in RASFs. IL-6 and soluble IL-6 receptor *α* (sIL-6R*α*) increased the expression of MMP-1, MMP-3, and MMP-13, but not MMP-9. IL-6-induced transcription factor signal transducer and activator of transcription 3 (STAT3) were found to bind to the MMP-1, MMP-3, and MMP-13 promoters, but not the MMP-9 promoter. High expression of IL-6 was associated with high levels of acetylation at H3 (H3ac) in the IL-6 promoter in RASFs [88]. Curcumin, a HAT inhibitor, decreased IL-6 expression and the level of H3ac in the IL-6 promoter in RASFs. Huber et al. reported that nuclear HDAC activity was low in RA synovial tissues, whereas nuclear HAT activity was similar in RA and osteoarthritis (OA) synovial tissues [89]. Expression of HDAC1 and HDAC2 was repressed in RA synovial tissues. It is suggested that the balance between HAT and HDAC activities shifted to histone hyperacetylation in RA. On the other hand, Kawabata et al. showed that nuclear HDAC activity increased in RA synovial tissues and was associated with the amount of cytoplasmic TNF*α* [90]. HDAC1 is highly expressed in RA synovial tissues, and its activity and expression are upregulated after TNF*α* stimulation. In view of conflicting data on the roles of histone acetylation-catalyzing enzymes in RA, additional studies are thought to be needed. IL-6 and IL-8 expression was reduced in RA synovial tissues by HDAC inhibitors (HDACi), including trichostatin A (TSA), sodium phenylbutyrate, and nicotinamide [91]. In addition, HDACi, such as TSA and givinostat, repressed IL-6 production that was induced by IL-1*β*, TNF*α*, and Toll-like receptor (TLR) ligands and also decreased the stability of IL-6 mRNA in RASFs [92].

### 4.2. DNA Methylation in RASFs

Several reports have suggested the contribution of DNA methylation to the pathogenesis of RA, and a variety of altered DNA methylation patterns have been described in RASFs (Table 2). Global genomic DNA hypomethylation was seen in RA synovial tissues [93]. Proliferating RASFs were deficient in DNMT1 and 5-azacytidine (5-azaC), an inhibitor of DNMTs, provided normal SFs with the activated phenotype of RASFs. DNA hypomethylation upregulated expression of 186 genes, including growth factors/receptors, extracellular matrix proteins, adhesion molecules, and matrix-degrading enzymes. In spite of similar levels of DNMT1 transcripts between RASFs and OASFs [94], DNMT1 protein expression is reduced in RASFs, in particular upon stimulation with cytokines or growth factors [93]. In addition, IL-1 stimulation decreases DNMT1 transcription. Death receptor 3 (DR3) is a member of the apoptosis-inducing Fas gene family. Enforced hypermethylation of the CpG island repressed DR3 gene expression, which resulted in resistance to apoptosis in RASFs [95]. Differentially methylated genes between RASFs and OASFs were examined by methylated DNA immunoprecipitation and promoter tiling assays, which showed that TBX5 is less methylated in RASFs than in OASFs [86]. TBX5 induces the production of chemokines, such as IL-8, CXCL12, and CCL20. Basal expression of CXCL12 was high in RASFs and low CpG methylation was found in the CXCL12 promoter of RASFs [96]. 5-azaC increased CXCL12 expression and decreased the methylation of CpG nucleotides in the CXCL12 promoter of RASFs. Furthermore, genome-wide analyses of DNA methylation loci in RASFs were performed. Nakano et al. reported 1859 differentially methylated loci [97]. Some of hypomethylated loci were key genes in the pathogenesis of RA, including CHI3L1, CASP1, STAT3, MAP3K5, MEFV, and WISP3. TGFBR2 and FOXO1 were identified as hypermethylated loci. Pathway analysis showed that hypomethylated genes were related to cell migration, cell adhesion, transendothelial migration, and extracellular matrix interactions. Whitaker et al. determined whether DNA methylation signatures change in long-term cultured RASFs [98]. The genome-wide patterns of differential DNA methylation of RASFs were examined at passages 3, 5, and 7 and were quite similar regardless of passage number. By analyses of pathway and ontology databases, differentially methylated genes were associated with innate immunity, cell adhesion, and cytokines.

### 4.3. miRNAs in RASFs

Several miRNAs are associated with the pathogenesis of RA (Table 3). For example, the basal expression level of miR-34a^*∗*^ is repressed in RASFs [99]. The promoter of miR-34a^*∗*^ is methylated and the transcription of miR-34a^*∗*^ increases upon treatment with 5-azaC. X-linked inhibitor of apoptosis protein (XIAP) was identified as a direct target of miR-34a^*∗*^. XIAP blocks apoptosis by direct binding to caspases. Enforced expression of miR-34a^*∗*^ caused Fas ligand- (FasL-) and TNF-related apoptosis-inducing ligand- (TRAIL-) mediated apoptosis in RASFs. Downregulation of proapoptotic miR-34a^*∗*^ resulted in upregulation of XIAP, thereby contributing to the resistance of RASFs to apoptosis. Alterations in the expression of miRNAs in RASFs were examined by screening 260 miRNAs [100]. The expression of miR-203 was high in RASFs, and 5-azaC upregulated miR-203 expression. Enforced expression of miR-203 increased production of MMP-1 and IL-6. Upregulation of IL-6 by miR-203 overexpression was repressed by inhibition of the NF-*κ*B signaling pathway, and basal IL-6 expression was correlated with basal expression of miR-203. Microarray analysis of miRNAs that were expressed in RASFs revealed that the expression of both miR-155 and miR-146a was constitutively high in RASFs [101]. The expression of miR-155 was upregulated after stimulation with TNF*α*, IL-1*β*, lipopolysaccharide (LPS), poly(I-C), and bacterial lipoprotein (BLP). Enforced expression of miR-155 in RASFs inhibited MMP-3 expression and repressed the induction of MMP-1 and MMP-3 by TLR ligands and cytokines. In another study, differentially expressed miRNAs in RASFs were screened by microarray analysis, which showed that miR-155 expression was also upregulated and induced by TNF*α* in RASFs [102]. Enforced expression of miR-155 reduced MMP-3 expression and inhibited the proliferation and invasion of RASFs. Inhibitor of kappa light polypeptide gene enhancer in B cells, kinase epsilon (IKBKE), is a target of miR-155, and it has been suggested that miR-155 may be a protective factor against inflammation by attenuating expression of IKBKE in RASFs. miR-22 directly targeted the 3′-UTR of Cysteine-rich angiogenic inducer 61 (CYR61) mRNA and repressed CYR61 expression [103]. CYR61 promotes RASF proliferation and differentiation of T helper 17 (Th17) cells that play an important role in the pathogenesis of RA. Expression of miR-22 was reduced and was negatively correlated with CYR61 expression in RASFs. Wild-type p53 induced miR-22 transcription by binding to the promoter of the miR-22 gene, whereas the mutant forms of p53 that were frequently observed in RASFs suppressed miR-22 expression. Stimulation of RASFs with LPS and BLP decreased miR-20a expression [104]. This decrease was associated with upregulation of apoptosis signal-regulating kinase (ASK) 1 that was a key component of the TLR4 pathway. ASK1 is a direct target of miR-20a. Overexpression of miR-20a decreased ASK1 expression in LPS- and BLP-activated RASFs. MicroRNA microarray analysis demonstrated that miR-19b was downregulated in RASFs [105]. miR-19b targets TLR2 mRNA and overexpression of miR-19b decreases expression of TLR2, IL-6, and MMP-3. It is thought that miR-19b can act as a negative regulator of inflammation in RA.

## 5. Conclusion

Increasing evidence has shown that aberrant epigenetic changes contribute to the development of RA and affect disease susceptibility and severity in RA. Further study is needed to reveal the crosstalk among these different epigenetic mechanisms in different cell types in RA. Synoviocytes are comprised of fibroblasts and macrophages, and not only SFs but also synovial macrophages are involved in inflammation of the RA synovium. Therefore, it will be important to investigate the relationship between SFs and synovial macrophages in RA, including how they can influence each other by epigenetic mechanisms. It is hoped that advances in the studies of the epigenetic mechanisms in RA will provide a better understanding of the pathogenesis of RA and help develop new therapeutic strategies and biomarkers for RA.

## Figures and Tables

**Table 1:** Abnormality of histone modifications in RASFs.

Epigenetic alterations	Function	References
Increase of H3K27me3 by upregulated EZH2	Decrease of SFRP1 involved in Wnt signaling inhibition	[85]
Increase of H3K4me3, decrease of H3K27me3, and increase of H3ac	Increase of TBX5 involved in chemokine production	[86]
Increase of H3K4me3 and decrease of H3K27me3	Increase of MMP-1, MMP-3, MMP-9, and MMP-13 involved in extracellular matrix degradation	[87]
Increase of H3ac	Increase of IL-6 involved in inflammation	[88]
Decrease of HDAC activity and expression	Histone hyperacetylation	[89]
Increase of HDAC activity and expression	Histone hypoacetylation	[90]

**Table 2:** Abnormality of DNA methylation in RASFs.

Epigenetic alterations	Function	References
Global genomic hypomethylation and decrease of DNMT1 protein expression	Increase of 186 gene expressions	[93]
Normal DNMT1 gene expression	Decrease of DNMT1 gene expression after IL-1 stimulation	[94]
Hypermethylation	Decrease of DR3 involved in resistance to apoptosis	[95]
Hypomethylation	Increase of TBX5 involved in chemokine production	[86]
Hypomethylation	Increase of CXCL12 involved in inflammation	[96]
Genome-wide differential methylation	1859 differentially methylated loci	[97]
Genome-wide differential methylation	2375 differentially methylated loci	[98]

**Table 3:** Abnormality of miRNAs in RASFs.

miRNA	Expression change	Function	References
miR-34^*∗*^	Downregulation	Increase of XIAP involved in resistance to apoptosis	[99]
miR-203	Upregulation	Increase of MMP-1 involved in extracellular matrix degradation Increase of IL-6 involved in inflammation	[100]
miR-155	Upregulation	Decrease of MMP-3 involved in extracellular matrix degradation	[101]
miR-155	Upregulation	Decrease of IKBKE involved in inflammation	[102]
miR-22	Downregulation	Increase of CYR61 involved in cell proliferation and Th17 cell differentiation	[103]
miR-20a	Downregulation	Increase of ASK1 involved in TLR4 pathway	[104]
miR-19b	Downregulation	Increase of TLR2 involved in innate immunity	[105]
